# Who Is Responsible?

**Published:** 1897-10

**Authors:** 


### EDITORIAL DEPARTMENT.

F. E. DANIEL, M. D., Editor.
S. E. HUDSON, M. D., Managing Editor.
A. J. SMITH. M. D., Galveston, Associate Editor.

Published Monthly at Austin, Texas, by Drs. Daniel and Hudson. Subscription price $2.00 a year in advance.

Eastern Agents: The Monihan-Fairchild Special Agency, St. Paul Building, New York City.

Official organ of the West Texas Medical Association, the Houston District Medical Association, the Austin District Medical Society, Brazos Valley Medical Association, the Galveston County Medical Society, and several others.

### WHO IS RESPONSIBLE?

The responsibility of permitting yellow fever to gain entrance to our shores at Ocean Springs and Boloxi, Miss., rests clearly upon the U. S. Marine Hospital Service.

It will be remembered that some while back, when the Mississippi State Quarantine Station on Mississippi Sound was destroyed by storm, the Marine Hospital Service, which maintains, and for years has maintained, a station on Ship Island, proposed to the Mississippi State Board of Health to relieve them of the expense and trouble of guarding the coast, telling them that it was altogether unnecessary to rebuild their station. The offer was accepted; but within a year the Mississippi health authorities became alarmed at what they denominated the "lax methods" of the Marine Hospital Service, and declared that Mississippi could not afford to take the risks that this service was permitting. Accordingly, therefore, the State Board rebuilt their station and resumed the function of State quarantine. But a conflict soon occurred between the two authorities, and the Marine Hospital Service having the backing of the U. S. Government, knocked out the State Board at one blow. An order from the Treasury Department was secured forbidding vessels to sail on the certificate given by the State Board. This, of course, paralized the State authorities, and although their officer is kept on duty on the coast, the Marine Hospital Service is known to be in charge, and they are, therefore, responsible.

Ship Island, the seat of the Federal quarantine, and solely in charge of U. S. Marine Hospital Service officers, is a few miles out from the coast line. It is the "refuge station" where all badly-infected vessels sent from quarantine stations are treated.

It is, therefore, necessarily infected, and is doubtless the worst infected and most dangerous spot on the coast anywhere. The shell banks around about Ship Island are reported to be the best fishing grounds, and we learn that Ship Island is a place of general resort for fishing and picnic parties. There is not a shadow of doubt that the infection was in this way carried to the shore.

The surgeons and assistant surgeons of the Marine Hospital Service, who are stationed at such places, are for the most part men who have only a "theoretical knowledge" of yellow fever, there being in the service only three or four yellow fever experts, and these are on duty as experts; are called in, when in doubt, to make a diagnosis, too often, alas, after the disease has gotten in its deadly work.

If, therefore, it was because of want of practical knowledge of the disease and its mode of propagation on the part of those in charge at Ship Island, that the infection was permitted to get to the shore, the responsibility is none the less great. If it were due to negligence or want of proper care, it is criminal. In any event, that the disease escaped from the hands and control of the Marine Hospital Service is a fact deserving the severest condemnation.

The importance of this matter, as to responsibility, is greatly intensified when it is recalled that the Marine Hospital Service has, without warrant in law, and upon a technicality, seized upon and now holds one of the Texas quarantine stations, over the protest of Governor, State Health Officer and the people of the vicinity. A comparatively young man, inexperienced in treating yellow fever, and who is on record for having made a false diagnosis of the cases on the Steamship Helen in 1895—pronouncing cases on that vessel to be yellow fever when they were not—is in charge at Sabine Pass, and has superceded the veteran quarantine officer and yellow fever physician, Dr. Perkins, at that place. Can the people of Texas feel the same security they have felt all these years when that port was guarded by a man of ripe experience and mature judgment, and who, in addition to his well known capability and conscientious regard for and performance of duty, has, at that post, everything on earth that is dear to him to protect—his home, his wife and children—some of whom, probably, were born there? Would the citizens, therefore, be much to blame if, in knowledge of the fact that the present epidemic is the third one that has been introduced into the South through the ignorance, neglect or incompetence of the Marine Hospital officials, and the further fact that this service has unlawfully wrenched control from the hands of our tried and trusted officer, they should, in their sense of insecurity, resort to the less scientific but doubly sure methods of former days, and should bring the shotgun into requisition?

It is a matter of deep and serious importance to the people of Texas. If the State authorities were not thus interfered with, they would, I feel assured, protect the gulf coast and all other points, against the introduction of the disease, as small as is the amount of money at the disposal of the Health Officer; but his appointee has been set aside, and Dr. Magruder, of the Marine Hospital Service, is the responsible party at one of our danger ports. Should yellow fever slip by him at Sabine Pass, as it evaded the Marine Hospital Service at Brunswick, Ga., in 1893, and at Boloxi, Miss., in 1882, and at Ocean Springs in 1897, and devastate the interior, the people must fix the responsibility where it belongs.

Whether we are to escape a general epidemic of yellow fever, or whether the State is to be scourged, as it was in 1867 and 1873, remains to be seen. In either event, it is hoped that the lesson will not be lost. The State of Texas had so long enjoyed exemption from epidemics under the able and enlightened administration of our "one man system of quarantine," as certain parties have called it, that the people were beginning to feel perfectly secure, and a sentiment was being actively cultivated that quarantine was a kind of expensive luxury, something entirely unnecessary, and in a few years more of safety, the legislature would, very probably, have voted to dispense with it. Now, they are again reminded that quarantine is a necessity, and that it takes money to run it. There are no funds at the disposal of the Public Health Department of Texas to meet this contingency, and the State Health Officer will be compelled to use the small amount appropriated for quarantine purposes—just sufficient to pay known expenses in times of peace. That there will be a deficiency is absolutely certain.

In vain was the legislature asked to set aside an amount for contingencies like this. In vain was it represented to them that the sum of $33,000 was barely sufficient to keep up the regular stations in times of peace, and that, should we be threatened with yellow fever or cholera, money would be needed for additional stations and medical officers and guards; and that, if it should not be needed, it would not be used; but if needed, it would be like the fellow and his pistol, it would be needed mighty bad! There was no use in talking. Old gray-haired senators, who ought to have remembered the horrors of 1867 and 1873, were the most bitter opponents of any appropriation at all for quarantine.

---

Dr. G. L. Robertson, of Leander, Texas, reports to the Journal an interesting case in which an abscess formed in Douglas' sac several days after delivery of a dead child. It was an arm presentation. The doctor succeeded in replacing the arm, and bringing down the feet, delivered without much trouble. There was considerable shock and hemorrhage. The uterus was irrigated by the use of hot water and peroxide of hydrogen. In two days there came on fever, and the womb was again cleansed, a second time there were rigors of a severe nature and a high temperature. He was at a loss to account for this, and searched for a cause. On examination, per rectum, he found "in Douglas' sac,—extending up to the posterior wall of the uterus and along the ligament to the right ovary, a collection of pus." When this had been evacuated by an incision, and the parts thoroughly cleansed, the temperature fell to normal, and the patient made a good recovery; there being no adhesions, the womb was freely movable.

---

The appointment of Dr. J. W. McLaughlin, of Austin, to the Chair of Theory and Practice of Medicine in the Medical Department of the University of Texas, vice Prof. West, resigned, will be hailed with general satisfaction by the medical profession of Texas. It is eminently fitting and proper. Dr. McLaughlin is universally esteemed by his confreres as a ripe scholar and a profound and original thinker. Personally he is very popular. He brings to the Chair a large and varied experience gleaned in an active general practice of over twenty-five years—a fine presence, a ready command and easy flow of language, and an aptness at imparting to others his ideas and views. More than that, he will bring the support and good will of almost the entire profession. His appointment will do much toward cementing their good will and influence, which have been somewhat alienated heretofore by the action of the Regents in sending to Europe for teachers which they feel and believe could have been found in Texas equally able and efficient. The JOURNAL is especially pleased at the selection. It could not have fallen upon one more worthy the honor or capable of filling the position with credit to himself and satisfaction to his constituents. The citizens of Austin gave Dr. McLaughlin a reception and presented him with a handsome silver service on the eve of his departure for his new field, and congratulations were mingled with regrets at losing him to Austin.

